# Alphabetism and the science of reading: from the perspective of the akshara languages

**DOI:** 10.3389/fpsyg.2014.00866

**Published:** 2014-08-12

**Authors:** Sonali Nag

**Affiliations:** ^1^Early Childhood and Primary School Programmes, The Promise FoundationBangalore, India; ^2^Department of Experimental Psychology, University of OxfordOxford, UK

**Keywords:** akshara, orthographic processing, cross-linguistic, alphasyllabary, hindi

An interesting area of enquiry in reading science is the ways in which different writing systems represent language. Discussions have centered around the adaptations seen between writing systems and languages (Perfetti and Harris, 2013) and the related notions of deservedness of a writing system for a language (Halliday, 1977), optimality (Frost, 2012) and level of orthography-language or “grapholinguistic” equilibrium (Seidenberg, 2011). Among the many ideas of relative goodness of writing systems is also a misplaced superiority assigned to alphabet-based orthographies, which has been critically labeled as “alphabetism” (Share, 2014). Share counters the superiority claim with psychoacoustic, historic, anthropological and preliminary experimental evidence to show that syllable-based writing systems are perhaps the better system, at least for some aspects of the orthography-language relationship. The defining parameters for placing symbol systems in a hierarchy are however, as yet, unclear (see Frost, 2012 for a discussion). It is for this very reason that reading research (and the practice it influences) must be alert to unqualified generalizations made from studies conducted in a single writing system. Evidence from robust cross-orthographic experimentation is the best moderator of such universalism. The burgeoning body of work from the Chinese languages has for example broadened the field, and perhaps snuffed out “alphabetism” in some domains (e.g., neural bases of reading and the preferred ordering of symbols as linear: Perfetti et al., 2010). Some insights are now also available from experimental work and surveys in Japanese Hiragana (e.g., Fletcher-Flinn et al., 2014). More recently, research in the Indic alphasyllabaries highlights the role of orthography-specific investigations in the quest for a more inclusive reading science (Nag, 2007, 2014).

The orthographies of South and Southeast Asia descend from the ancient script of Brahmi and together may be referred to as the Indic alphasyllabaries. The symbol unit of these orthographies is the *akshara*. The surface organization of each unit is typically a symbol block with one or more phonemic markers. An akshara may represent a vowel (/V/), a consonant (/C/), a consonant with the inherent vowel /a/ or other marked vowels (/Ca/, /CV/), and consonant clusters with either the inherent or marked vowels (e.g., /CCa/, /CCV/, /CCCV/). The mapping of word level phonology to specific akshara is decided by a rule of re-syllabification where post-vocalic consonants form the next akshara. To illustrate with number names from the Indo-Aryan language of Hindi, the akshara in *shunya* (zero) follow the rule of re-syllabification with the second akshara formed by a coda-open syllable concatenation (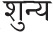, <CV.CCa> *“shu.nya*,” the coda of the first syllable is pinned to the next syllable to make the symbol block “*nya”*). The transcription in the akshara system is typically complete, though mapping to phonology is variable. For example, *nau* (nine) represents an open syllable (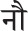, <CV>, “*nau”*), *das* (10) a body and coda (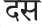, <CV.C°>, “*da.s”*), and *gyaarah* (11) an open syllable, a body and a coda (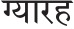, <CCV.Ca.C°>, “*gyaa.ra.h*”). There are further conditional rules in Hindi such as vowel suppression where the akshara-to-phonology representation becomes somewhat opaque. Thus, in *bees*, *thees*, and *chalees* (20, 30, and 40) the word-final /s/ is written with an akshara carrying the inherent vowel /a/ but this vowel is suppressed in pronunciation (i.e., <C°>), thus 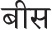 and 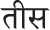, <CV.C°>, and 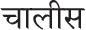, <CV.CV.C°>. Similar schwa suppression is also seen in the earlier examples, *das* and *gyaarah*.

Akshara-based orthographies such as Bengali, Gujarati, Lao, Tamil, and Sinhala each have similarly well-defined orthographic principles. Whereas in other phonologically-based writing systems like the alphabet and the abjad, the orthographic representation of one particular sub-lexical level predominates, the mapping to phonology in the akshara-based orthographies is defined by context. If appearing single, then the akshara is typically an orthographic syllable, but if in a string, language-specific rules delimit orthographic representation. Thus, akshara units map to multiple levels of phonology. Given the current state of the science, this psycholinguistic design of the akshara requires greater examination. But what should be immediately clear is that the pre-eminence given to the phoneme in several accounts of orthographic representation (e.g., Katz and Frost, 1992; Ziegler and Goswami, 2005) is an alphabet-centric model. The akshara based psycholinguistic tradition has instead drawn upon the role of orality in literacy development (Patel and Soper, 1987; Patel, 1996, 2004), the articulatory features of single akshara and word-level prosody (Pandey, 2007, 2014), the nature and scope of akshara-language mapping (Sircar and Nag, 2013; Nag, 2014), the cognitive bases of reading acquisition (Prakash et al., 1993; Nag and Snowling, 2012) and the profiles of impairment in adult clinical conditions (Karanth, 2002). What is needed for a universal theory of reading (and spelling) development is a delineation of the cognitive-linguistic mechanisms associated with a writing system that has the facility for multiple levels of sub-lexical representation. Constructs that have shown promise include syllable weight and the *mora*. These constructs pick out the regularities in spelling-sound mapping and hence may be the principle that makes learning of the orthography-language connections secure. Ideas about syllable weight and the *mora* have deep roots in linguistic science but are yet to inform discourse in the reading science.

The symbol set is another case in point. The number of letters in alphabet-based systems is small, and symbol learning is completed within the first year of instruction. In contrast to the small set or a *contained orthography*, are systems with several thousand symbols. The characters for a Chinese language such as Mandarin is one example of an *extensive orthography*. In the Indic alphasyllabaries, the number of akshara that can be hypothetically constructed also run into thousands, with two constraints defining the learning space. First, a manageable set of consonant and vowel phonemic markers aid akshara construction, bringing economy to the learning task. Second, the number of akshara that are phonotactically implausible are far more in number, although the number that are in use and hence encountered in print still runs into hundreds. Not surprisingly, a corollary of an extensive symbol set is that symbol learning continues well into middle school and beyond. If the received wisdom is that children typically always know the alphabet by the end of the first year then it is not hard to see how the pace of learning in the extensive orthographies might be perceived. “Slow” learning then becomes one reason to invoke “alphabetism,” with suggestions that the local orthographies are too difficult for fast paced literacy learning.

Furthermore, a comprehensive theory of literacy learning will have to factor in the learning mechanisms involved in the akshara languages, particularly the role of domains such as visual memory, morphology and syntax, and several other aspects of the orthography. Some of these include non-linear symbol arrangements (Vaid and Gupta, 2002; Kandhadai and Sproat, 2010; Winskel and Perea, 2014), unmarked and inherent symbol features (Nag, 2007; Bhide et al., 2014), visually complex symbol sets (Nag et al., 2014) and word types differing because of symbol characteristics (Nag, 2014; Wijayathilake and Parrila, 2014) or morpho-orthographic characteristics (Rao et al., 2012). A step before the hunt for higher-order universals would be to bring focus in reading science on these kinds of particularities.

## Conflict of interest statement

The author declares that the research was conducted in the absence of any commercial or financial relationships that could be construed as a potential conflict of interest.
